# Measurements for the Development of a Simulated Naturally Occurring Radioactive Material

**DOI:** 10.6028/jres.117.008r2012

**Published:** 2012-04-01

**Authors:** L. Pibida

**Affiliations:** National Institute of Standards and Technology, Gaithersburg, MD 20899-8462

**Keywords:** Naturally occurring radioactive materials, NORM content, NORM gamma-ray spectra, simulated NORM

## Abstract

Nineteen different commercially available samples containing naturally occurring radioactive materials (NORM) (i.e., natural uranium, thorium, radium and potassium) were investigated, including zircon sand, cat litter, roofing tiles, ice melt and fertilizer among others. A large variation in isotopic composition was observed across the measured samples. As a result of this observation, a need was identified to develop and implement the use of a *simulated NORM sample* to serve as a reference standard sample containing naturally occurring radioactive elements. The purpose of the simulated NORM sample would be to simulate typical samples containing NORM to be used for testing radiation detection instruments against ANSI/IEEE and IEC document standards requirements. The design and construction of the proposed new simulated NORM sample and the subsequent energy spectra characterization measurements are presented as part of this work.

## 1. Introduction

The crust of the earth is composed of a great variety of elements. Some of these elements emit gamma-ray radiation that can be detected by radiation detection instruments designed to detect man-made radioactive sources. These elements are referred to as naturally occurring radioactive materials (NORM) and are mainly natural uranium, thorium, radium and potassium. Maps showing the concentration of these elements in the United States and Canada can be found at the United States Geological Survey web site [1]. Several commodities, such as roofing tiles, cat litter and ice melt, contain NORM due to the materials used in their production. Therefore, NORM is present in commerce worldwide. The presence of NORM creates a challenge for radiation detection instruments, used in a variety of applications to detect man-made radioactive sources, currently deployed in many locations around the world [2,3]. NORM materials can trigger undesired (nuisance) radiation alarm signals in radiation detection instruments resulting in a false detection or indication of radiation from man-made sources.

Radiation detection instruments with gamma-ray spectrometric capabilities can identify radionuclides present in NORM and discriminate them from those present in different man-made radioactive sources. However, the discrimination is sometimes limited by the amount of NORM. In addition, the discrimination can be limited by the degree of overlapping of the NORM gamma-ray emission lines with those from the man-made radioactive sources. Several documentary standards, including those published by the American National Standard Institute/The Institute of Electrical and Electronic Engineers (ANSI/IEEE) and International Electrotechnical Commission (IEC), set minimum performance requirements for NORM discrimination for radiation detection instruments with gamma-ray spectrometric capabilities [4–10]. Parts of these requirements include the ability of detectors to identify target radioactive sources, mainly ^235^U and ^239^Pu, when in the presence of NORM.

In this report, a summary is presented for measurements performed with different types of samples containing NORM referred to hereafter as *NORM samples*. The NORM samples used here are considered to be a good representation of products found in commerce that contain different amounts of natural uranium, thorium, radium and potassium. Nineteen different commercially available NORM samples were measured, including zircon sand, cat litter, roofing tiles, ice melt and fertilizer among others. A large variation in isotopic composition was observed across the measured NORM samples. As a result of this observation, a need was identified to develop and implement the use of a *simulated NORM sample* to serve as a reference standard sample containing naturally occurring radioactive elements. The purpose of the simulated NORM sample would be to simulate typical NORM samples that are commercially available (e.g., zircon sand, cat litter, roofing tiles, ice melt and fertilizer). Furthermore the *simulated NORM sample* would be required to have a well-known isotopic composition for testing detectors against ANSI/IEEE and IEC documentary standards. Testing of radiation detection instruments are typically conducted at different laboratories. One of the conditions for conducting such tests is to ensure that NORM masking test results are reproducible across different testing facilities. In this work, measurements were conducted to develop a simulated NORM sample that would allow testing laboratories to have a standard sample, leading to reproducible test results. For this purpose, multiple energy spectra measurements were performed using ^226^Ra and ^232^U (used to replace ^232^Th) point sources shielded by different materials in order to simulate a bulk spectrum and keep isotopic ratios similar to some of the measured NORM samples that are commercially available (i.e., cat litter, zircon sand). Measurements were performed using several high purity germanium (HPGe) detectors.

## 2. Experimental Setup

Measurements were performed using five closed-end coaxial HPGe detectors. These detectors were set up according to specifications described in the ANSI/IEEE N42.14 standard [11]. Efficiency curves for all of these detectors were generated by acquiring photon data from several radioactive point sources. The radioactive point sources were prepared from NIST standard reference materials (SRM), and consist of radionuclides emitting gamma-rays with energies between 35 keV and 2.6 MeV. The relative combined standard uncertainties in the efficiency measurements are less than 1.5%. The radioactive point source activities used for the efficiency measurements ranged from 10^3^ Bq to 10^7^ Bq depending on the source-to-detector distance for the calibration geometry. The half-lives, gamma-ray emission probabilities, and uncertainties used for the detector efficiency calibrations are those listed in the Evaluation Nuclear Structure Data File (ENSDF) tables available from the National Nuclear Data Center [12] or from the Laboratorie National Henri Becquerel [13]. The reference radioactive point sources used for calibration included ^54^Mn, ^60^Co, ^67^Ga, ^88^Y, ^109^Cd, ^113^Sn, ^125^I, ^133^Ba, ^137^Cs, ^139^Ce, ^210^Pb, ^203^Hg, ^207^Bi, ^232^U, and ^241^Am, and the combined standard uncertainties associated with the source activities ranged from 0.1% to 0.6% (k = 1), depending on the radionuclide.

Efficiency transfer calculations for the different measurement geometries used with all the NORM samples investigated were performed using the Efficiency Transfer of Nuclide Activity (ETNA) program [14]. Spectral data was analyzed using the Genie-2000 [15] and FitzPeaks [16] programs^1^. For most of the NORM samples, the time to acquire an energy spectrum was one day. For a fewer number of NORM samples, the acquisition times were extended to 5 days to obtain more photon counts in each one of the relevant peaks of the energy spectrum to reduce the uncertainties in the measurements. The mass of the measured samples varied between 500 g and 30 kg. The mass of the smaller samples varied between 500 g and 3 kg, and the mass of the larger samples varied between 13 kg and 30 kg.

The source activity per unit mass, *A*, was calculated using equation (1)

(1) $$A=\frac{N\left(E\right)}{T\times M\times \varepsilon \left(E\right)\times P\left(E\right)}\prod_{i}C_{i}$$

where *N*(*E*) is the number of counts in the full-energy peak, *T* is the measuring time, *ε*(*E*) is the full-energy-peak efficiency, *P*(*E*) is the gamma-ray emission probability at the energy *E, M* is the mass of the sample and ∏*C_i_* is the product of the correction factors, *C_i_*, applied to the measurement. The only correction factors that apply to these measurements are those due to geometrical differences between the point source calibration and the NORM sample measurement geometries, including self-attenuation. Decay corrections were neglected due to the long half-life of the main NORM radionuclides (^40^K, ^226^Ra, ^232^Th, ^238^U). The uncertainty of the source activity per unit mass was obtained using uncertainty propagation and assuming that all measured quantities are independent. The uncertainty for the source activity per unit mass, *u_A_*, is given by

(2) $$u_{A}=\sqrt{{\left(\frac{\partial A}{\partial N}\right)}^{2}u_{N}^{2}+{\left(\frac{\partial A}{\partial T}\right)}^{2}u_{T}^{2}+{\left(\frac{\partial A}{\partial \varepsilon }\right)}^{2}u_{\varepsilon }^{2}+{\left(\frac{\partial A}{\partial P}\right)}^{2}u_{P}^{2}+{\left(\frac{\partial A}{\partial M}\right)}^{2}u_{M}^{2}}$$

where *u_N_*, *u_T_*, *u_ε_*, *u_P_*, and *u_M_* are the uncertainties associated with the quantities *N*(*E*), *T*, *ε*(*E*), *P*(*E*), and *M*, respectively. When source geometry corrections were included in the activity calculations, their associated uncertainties were added in quadrature with the other uncertainty components. For the calculation of the emission rate ratio, *R*, shown in Fig. 7, the uncertainty, *u_R_*, was determined by

(3) $$u_{R}=\sqrt{{\left(\frac{\partial R}{\partial R_{1}}\right)}^{2}u_{R_{1}}^{2}+{\left(\frac{\partial R}{\partial R_{2}}\right)}^{2}u_{R_{2}}^{2}}$$

where *R* is determined as the ratio of the emission rates of gamma-ray lines*, R*_1_ and *R*_2_, corresponding to two individual gamma-ray lines from the energy spectrum, and *u_R_*_1_, *u_R_*_2_, are the associated uncertainties. The main contribution to these uncertainties is the counting statistics.

The energy spectra for a large number of NORM samples were measured, including: slate, cat litter, ice melt, roofing tiles, hay, coal, fertilizer, Australian zircon sand, diammonium phosphate (DAP), ISG Pye, CEMEX type FC, monocalcium phosphate (biofos), allanite, monazite, pyrochlore and zircon. In addition, an investigation was conducted to develop a *simulated NORM sample*. For this purpose, energy spectra measurements were performed using 740 kBq ^232^U and 296 kBq ^226^Ra point sources paired together and shielded by different thicknesses of polymethyl methacrylate (PMMA). The ^226^Ra was placed in front of the ^232^U (between the ^232^U and the detector). This configuration ensured that the 186 keV line originating from the ^226^Ra source was being attenuated only by the PMMA shielding surrounding both sources. The point source encapsulation was described in reference [17]. The PMMA thickness was varied between 4 cm and 15 cm. The material from which the ^232^U point source originated is approximately 30 years old, so the gamma-ray emission is very close to that of a ^232^Th source.

## 3. Results and Discussion

**Fig. 1:**
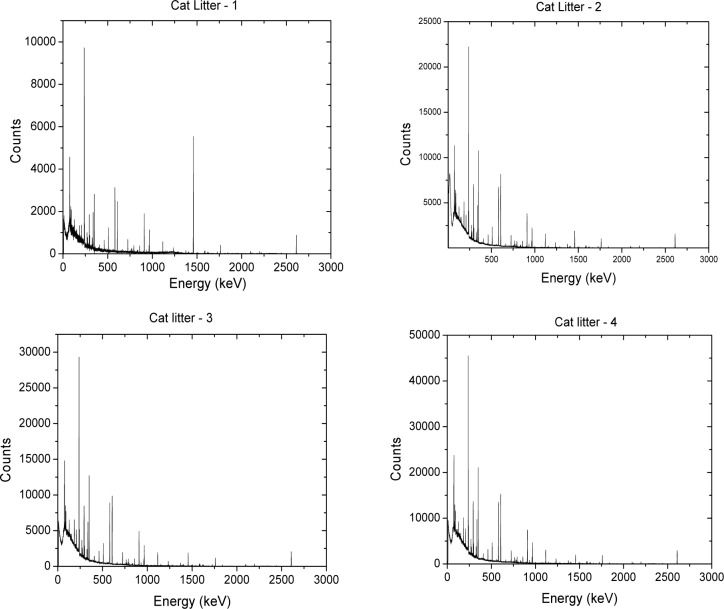
Energy spectra for four different brands of cat litter source samples.

**Fig. 2:**
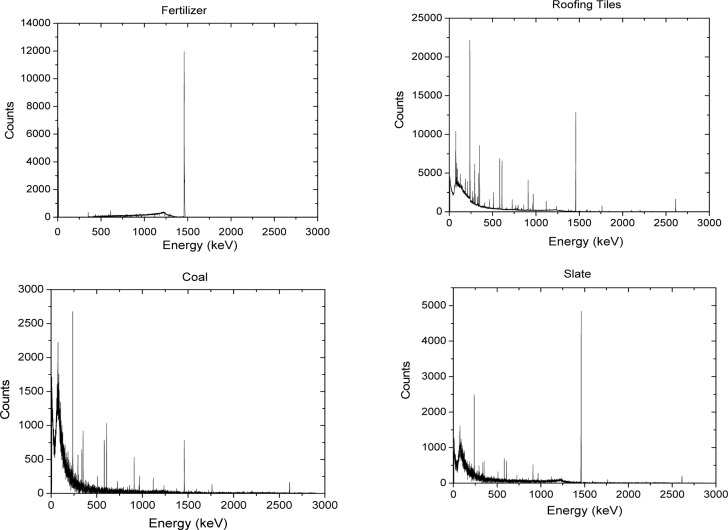
Energy spectra for the fertilizer, roofing tiles, coal, and slate source samples.

**Fig. 3:**
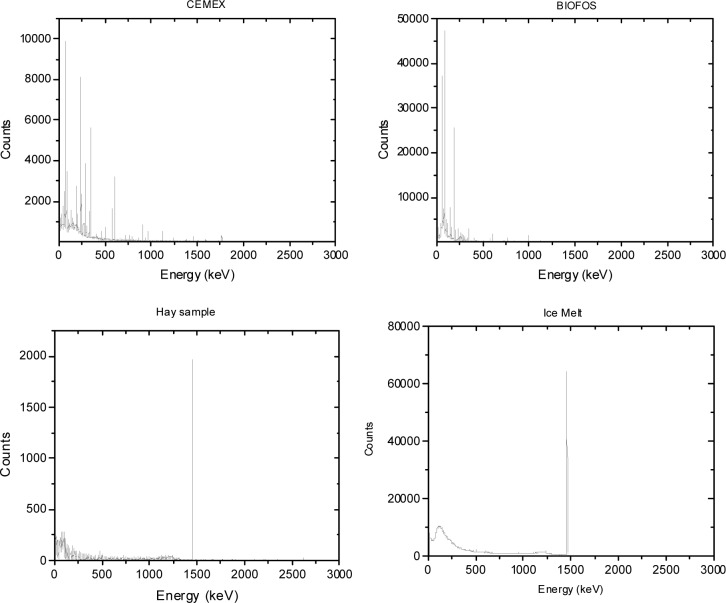
Energy spectra for the CEMEX, BIOFOS, hay, and ice melt source samples.

**Fig. 4:**
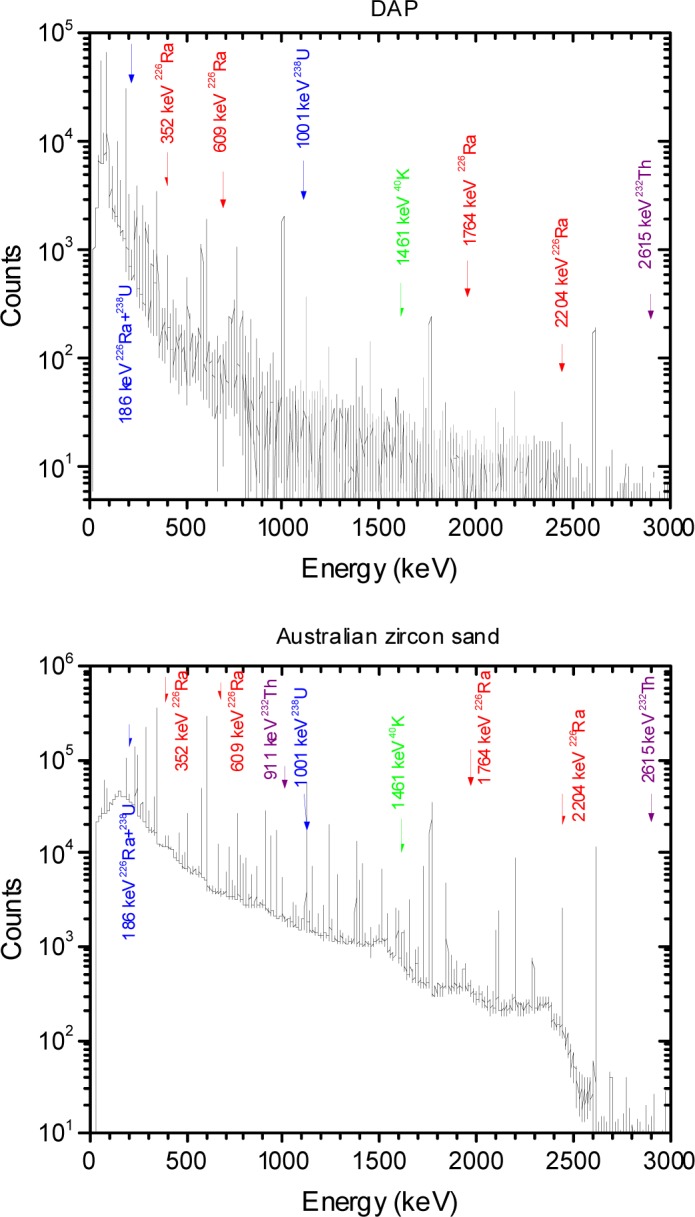
Energy spectra for the DAP and the Australian zircon sand source samples. The main gamma-ray lines are shown for both samples.

**Fig. 5:**
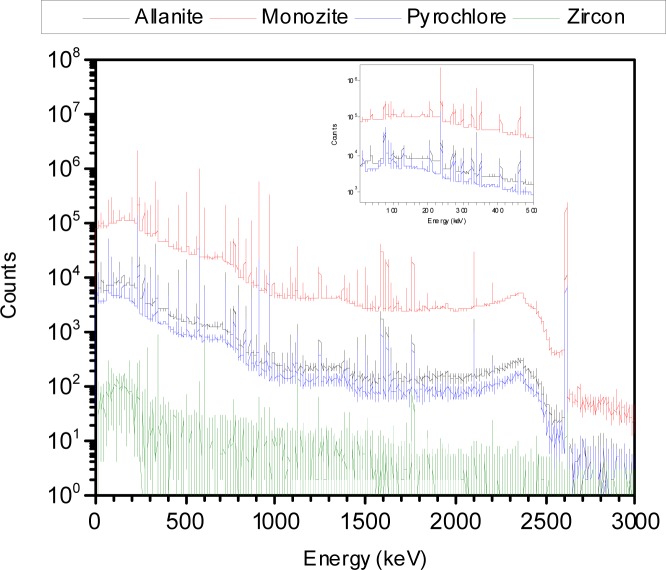
Energy spectra for the allanite, monazite, pyrochlore and zircon source samples. These were the smallest size samples.

The background subtracted energy spectra acquired for the different NORM samples tested are shown in Figs. 1–5. Table 1 and Table 2 summarize the measured activity per unit mass for some of the measured NORM samples. From these figures and tables it can be observed that the isotopic composition and source activity vary widely between the different NORM samples. Furthermore, a large isotopic variation is observed for samples of the same material belonging to different batches and/or brands (see for example in Table 1 the four cat litter sample compositions). This lack of reproducibility in the isotopic composition for a given type of material makes the use of commercially available NORM samples inadequate for testing detectors consistently across testing laboratories. To illustrate the problem, consider two testing facilities that separately purchase cat litter from the same or from different companies. Under the assumption that both facilities have the same NORM sample, they conduct the testing of radiation detection instruments at their respective facilities. One facility used cat litter-1 while the other used cat litter-2. As shown in Table 1, due to the significantly different isotopic composition, the testing of the detectors could lead to different results. But this would not be due to the radiation detection instrument response itself but due to the differences of the source isotopic composition.

**Fig. 6:**
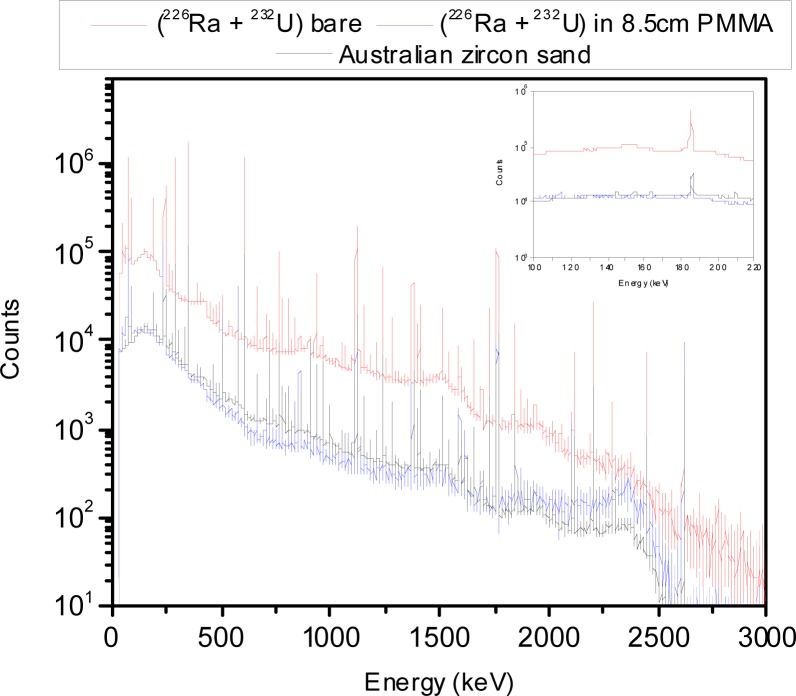
Energy spectra for the Australian zircon sand and the ^232^U point source together with ^226^Ra point source both bare and shielded by 8.5 cm of PMMA.

In order to address this problem, a *simulated NORM sample* was designed as part of this work. The simulated NORM sample was designed to generate a well-defined and reproducible energy spectrum. The construction parameters of the simulated NORM sample were chosen so that the energy spectrum closely matched the general characteristics of the spectra produced by the commercially available NORM samples listed in Table 1 and Table 2. In most of the ANSI/IEEE and IEC standards NORM is used to mask ^235^U and ^239^Pu sources so it is important to have a defined energy spectrum in the energy ranges of 186 keV and 320 keV to 420 keV and a contribution to the continuum that resembles bulk material. The ^40^K, with a gamma-ray line at 1460 keV, will only contribute to the continuum in the energy ranges of 186 keV and 320 keV to 420 keV. The ^238^U, with main gamma-ray lines at 186 keV and 1001 keV, will contribute to the continuum as well as to the 186 keV energy line. Therefore, the use of a combination of ^232^U and ^226^Ra point sources shielded by a material with a low-atomic number, such as PMMA, is appropriate to design the simulated NORM sample. Several source geometries were built and the energy spectrum for each of these source geometries was measured until optimum source geometry parameters were found. The various source geometries were achieved by varying the thickness of the PMMA shielding surrounding the pair of ^232^U and ^226^Ra point sources and comparing the measured spectra to those of the different NORM samples containing ^232^Th and ^226^Ra. For example, Fig. 6 shows the energy spectra for the Australian zircon sand sample and the pair composed of the ^232^U and ^226^Ra point sources both bare and shielded by 8.5 cm of PMMA. Spectra are normalized to the 2.6 MeV net peak areas. From Fig. 6 it can be observed that the contribution to the 186 keV gamma-ray line from the point source pair configuration is larger compared to that of the sand so additional shielding was added to reduce this contribution. The optimal PMMA thickness that best matched the net peak area for the 186 keV line observed in the Australian zircon sand was approximately 9.7 cm. From Fig. 6 it can also be observed that the continuum produced by the bare point sources is modified when the point sources are shielded with PMMA (8.5 cm thick), such that the continuum from the shielded point sources resembles the continuum produced by bulk material (in this particular case for the Australian zircon sand sample).

**Fig. 7:**
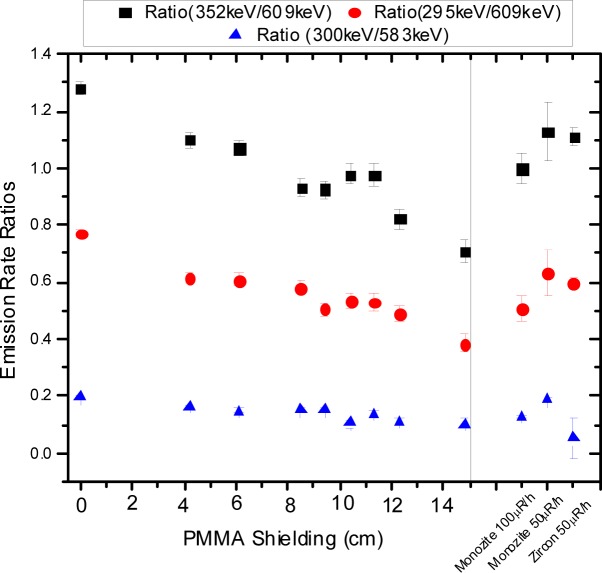
Ratios for main gamma-ray lines for the point sources as a function of PMMA thickness and three NORM samples. The exposure rate values produced by the NORM samples are measured and expressed in units of µR/h as required by the ANSI/IEEE standards [4–8]. The same monazite sample was measured at 2 exposure rates. The number in the numerator and denominator in the legend represent the energy of the gamma-ray lines. For example, the black squares represent the ratio of the emissions from the 352 keV and 609 keV gamma-ray lines. The uncertainty bars for each of the data points represent the uncertainty of the calculated ratios as explained in the text of the manuscript.

The ratios of the emission of different gamma-ray lines were derived from the measured energy spectra from all samples. In particular, the ratios were determined for the different sands listed in Table 1. Also, the ratios were obtained for the various source geometries built using different PMMA thickness values for the pair of ^232^U and ^226^Ra point sources. The values of the gamma-ray line emission ratios of these various geometry configurations using different PMMA thickness values were compared to three of the NORM samples as shown in Fig. 7. These ratios were obtained from the measured emission rate, for the main gamma-rays for the different radionuclides in the point source combination, using one of the calibrated HPGe detectors. The gamma-ray energies used to calculate these ratios were chosen to be between 295 keV and 609 keV so that the variations are not so sensitive to the variations in the PMMA thickness, like the case of the 186 keV line, or almost insensitive to the variations in PMMA thickness, like the case of the 2.6 MeV line. The gamma-ray energies were also chosen so that some belong to the ^226^Ra decay chain and others to the ^232^U decay chain. The gamma-ray lines belonging to the ^226^Ra decay chain are the 295 keV, 352 keV, and the 609 keV lines, those belonging to the ^232^U decay chain are the 300 keV and the 583 keV lines. In addition, the energy of these gamma-ray lines are close to the gamma-ray emission lines for ^239^Pu. From Fig. 7 it can be observed that the variation in the ratios is small for PMMA thickness values between 8 cm and 12 cm; for these thickness values the ratios also agree (within the uncertainties) with those obtained from some NORM samples producing different exposure rates at the reference point of the radiation detection instrument.

Based on these measurements, the optimal simulated NORM sample will have the ^232^U and ^226^Ra point sources surrounded by 9 cm of PMMA, while ensuring that the ^232^U source does not provide additional shielding to the ^226^Ra source (the point sources should be placed next to each other within the PMMA shielding material).

## 4. Conclusions

Due to the large variations of the source composition (i.e., activity and isotopic composition) found in commercially available NORM samples studied in this work, the use of *simulated NORM samples* is recommended. The design is based on the use of shielded radioactive point sources. The use of simulated NORM samples ensures the reproducibility of test results when testing radiation detection instruments against documentary standards. It was shown that the use of PMMA to shield paired ^232^U and ^226^Ra radioactive point sources produces similar energy spectra to those produced by samples containing NORM. The PMMA thickness can be adjusted such that the contribution of the 186 keV line from the ^226^Ra decay chain matches the spectral characteristics of an average NORM sample material.

## Figures and Tables

**Table 1:** Summary of the activity per unit mass measured for the different radionuclides that constitute some of the measured NORM samples

Sample	Radionuclide	Activity (Bq/kg)	Uncertainty (%)
Coal	K-40	207	30
	Ra-226	10	45
	Th-232	17	45
Roofing tiles	K-40	3518	30
	Ra-226	108	35
	Th-232	164	35
	U-238	4	45
Slate	K-40	1152	30
	Ra-226	4	40
	Th-232	16	30
	U-238	1	45
Hay	K-40	4409	30
Cat litter -1	K-40	1456	30
	Ra-226	58	35
	Th-232	100	35
	U-238	92	35
Cat litter - 2	K-40	408	30
	Ra-226	209	30
	Th-232	226	30
	U-238	380	30
Cat litter -3	K-40	468	30
	Ra-226	187	35
	Th-232	237	30
	U-238	198	35
Cat litter - 4	K-40	448	30
	Ra-226	404	30
	Th-232	434	30
	U-238	382	30
Ice melt	K-40	14607	30
Fertilizer	K-40	8889	35

**Table 2:** Summary of the activity per unit mass measured for the different radionuclides that constitute the smaller measured NORM samples

Sample	Radionuclide	Activity (Bq/kg)	Uncertainty (%)
Allanite	K-40	1397	35
	Ra-226	1155	35
	Th-232	17855	30
	U-238	2178	35
Monozite	Ra-226	24740	25
	Th-232	170903	25
Pyrochlore	Ra-226	1836	40
	Th-232	35303	30
	U-238	7833	35
Zircon	Ra-226	232	40
	Th-232	14	45
	U-238	219	40
ISG Pye	Ra-226	67	26
	Th-232	54	42
	K-40	151	48
DAP	Ra-226	15	29
	Th-232	10	34
	U-238	1923	35
	K-40	6	30
CEMEX type FC	Ra-226	57	28
	Th-232	39	27
	U-238	139	48
	K-40	60	30
Monocalcium phosphate (biofos)	Ra-226	16	23
	Th-232	11	43
	U-238	1970	31
	K-40	6	29
Australian zircon sand	Ra-226	1963	37
	Th-232	442	33
	U-238	3179	39

## Footnotes

1 Certain commercial equipment, instruments, or materials are identified in this paper to foster understanding. Such identification does not imply recommendation or endorsement by the National Institute of Standards and Technology, nor does it imply that the materials or equipment identified are necessarily the best available for the purpose.

## References

[b1-jres.117.008r2012] 1United States Geological Survey web site http://pubs.usgs.gov/of/2005/1413/maps.htm

[2] Kouzes RT, Ely JH, Geelhood RR, Lepel EA, Schweppe JE, Siciliano DJ, Strom DJ, Warner RA (2004). Naturally occurring radioactive materials and medical isotopes at border crossings. IEEE Nuclear Science Symposium-Conference Record.

[3] Burr Tom, Myers Kary (2008). Signatures for several types of naturally occurring radioactive materials. Applied Radiation and Isotopes.

[b4-jres.117.008r2012] 4ANSI/IEEE N42.34-2006, American National Standard Performance Criteria for Hand-held Instruments for the Detection and Identification of Radionuclides.

[b5-jres.117.008r2012] 5ANSI/IEEE N42.38-2006, Performance Criteria for Spectroscopy-Based Portal Monitors used for Homeland Security.

[b6-jres.117.008r2012] 6ANSI/IEEE N42.43-2006, Standard for Mobile and Transportable Systems Including Cranes used for Homeland Security Applications.

[b7-jres.117.008r2012] 7ANSI/IEEE N42.48-2008, American National Standard Performance Requirements for Spectroscopic Personal Radiation Detectors (SPRDs) for Homeland Security.

[b8-jres.117.008r2012] 8ANSI/IEEE N42.53-draft, Performance Criteria for Backpack Based Radiation Detector Systems Used for Homeland Security.

[b9-jres.117.008r2012] 9IEC 62327-2006: Radiation protection instrumentation - Hand-held instruments for the detection and identification of radionuclides and for the indication of ambient dose equivalent rate from photon radiation.

[b10-jres.117.008r2012] 10IEC 62484-2010: Radiation protection instrumentation - Spectroscopy-based portal monitors used for the detection and identification of illicit trafficking of radioactive material.

[b11-jres.117.008r2012] 11ANSI/IEEE N42.14-1999, American National Standard for Calibration and Use of Germanium Spectrometers for the Measurement of Gamma-ray Emission Rates of Radionuclides.

[b12-jres.117.008r2012] 12Evaluation Nuclear Structure Data File (ENSDF) table, National Nuclear Data Center web page http://www.nndc.bnl.gov/

[13] Laboratorie National Henri Becquerel Recommended Data.

[14] Lépy Marie-Christine, Bé Marie-Martine, Piton Francois (2001). Efficiency Transfer of Nuclide Activity (ETNA) program developed by the Bureau National de Métrologie (BNM/LNHB).

[b15-jres.117.008r2012] 15Genie-2000 Basic Spectroscopy Software Version 3.2.1, Canberra Industries.

[b16-jres.117.008r2012] 16FitzPeaks Gamma Analysis and Calibration Software Version 3.40, JF Computing Services, UK.

[17] Lucas L, Pibida L, Unterweger MP, Karam LR (2004). Gamma-ray Test Sources for Portal Monitors Used for Homeland Security. Radiation Protection Dosimetry.

